# Design and Implementation Factors for Performance Measurement in Non-profit Organizations: A Literature Review

**DOI:** 10.3389/fpsyg.2020.01799

**Published:** 2020-08-07

**Authors:** Fernanda T. Treinta, Louisi F. Moura, José M. Almeida Prado Cestari, Edson Pinheiro de Lima, Fernando Deschamps, Sergio Eduardo Gouvea da Costa, Eileen M. Van Aken, Juliano Munik, Luciana R. Leite

**Affiliations:** ^1^Industrial and Systems Engineering, Universidade Federal Tecnologica do Parana, Ponta Grossa, Brazil; ^2^Industrial and Systems Engineering, Pontificia Universidade Catolica do Parana, Curitiba, Brazil; ^3^Industrial and Systems Engineering, Virginia Tech, Blacksburg, VA, United States; ^4^DECIGI/PPGGI, Universidade Federal do Parana, Curitiba, Brazil; ^5^Industrial and Systems Engineering, Universidade Federal Tecnologica do Parana, Pato Branco, Brazil; ^6^DEMEC, Universidade Federal do Parana, Curitiba, Brazil; ^7^DEPS, Universidade do Estado de Santa Catarina, Florianópolis, Brazil

**Keywords:** non-profit organizations, performance measurement systems, literature review, bibliometric analysis, social network

## Abstract

**Purpose:** Performance measurement systems (PMS) in Non-profit Organizations (NPOs) are more complex than in for-profit organizations. NPOs have an orientation toward social mission and values, and they consider not only organizational efficiency and viability, but also the social impact of the organization. This research provides a comprehensive synthesis of PMSs in NPOs.

**Design/Methodology/Approach:** Using a literature review, supported by bibliometric and network analyses. A paper set of 240 articles related to this research field is examined. Topics that are the most prevalent in this research area and their interrelationships are identified, presenting an outline of current efforts.

**Findings:** Despite the descriptive analyses for the paper set, a framework is proposed for organizing the design-implementation factors of PMSs in non-profit organizations, identifying the main requirements for their successful development.

**Originality/Value:** Investigation on performance measurement in non-profit organizations is still in its early stages of development with many opportunities to further develop the field. Conceptual frameworks and models, as well as specific theories, are being generated for this field of research, and the process of adapting models from the general field of performance measurement is taking place. The meta-framework that organizes the main research topics of PMS in non-profit organizations and the framework that consolidates factors that influence the design-implementation of PMSs in non-profit organizations developed represents this paper contribution.

## Introduction

Social demands may be a challenge for governments and society. Non-profit organizations (NPOs) are an alternative approach to address collective needs of specific groups in the community and represent many types of organization, including universities, schools, hospitals, religious institutions, local, state and federal governments, non-governmental organizations (NGO), charitable institutions, trade unions, humanitarian aid agencies, foundations, cooperatives, civil rights organizations, political organizations and parties, and others that include volunteers and the third sector (Frumkin, 2005; Moxham, 2009, 2014; Valentinov, 2011).

According to Frumkin (2005), there are four basic functions of non-profit work: (i) service delivery, (ii) civic and political engagement, (iii) social entrepreneurship, and (iv) values and faith. The present paper is positioned in the supply and instrumental side of service delivery, where social goals are more important than profit and outcomes are measured by social value and social impact. Legally, an NPO has financial restrictions and cannot share profit. Profit is possible, but its use is restricted (Moxham, 2009, 2014; Kong, 2010; Valentinov, 2011).

Some Performance Measurement frameworks have been adapted for NPO and public administration. One of the most widespread PMSs in the literature and in practice is the Balanced Scorecard (BSC) developed by Robert Kaplan and David Norton and introduced in 1992. According to Hoque (2014), even when discussing other systems or frameworks developed since then, the BSC is broadly mentioned and sometimes used as a starting point. Despite that, Moxham (2009) and Straub et al. (2010) remark that this practice of adaptation is not so well-accepted.

Thus, this paper proposes a meta-framework that organizes the main research topics related to PMSs in non-profit organizations and, also, a framework to consolidate factors that influence the design-implementation aspects of PMSs in non-profit organizations. To accomplish this, a literature review was conducted. The main findings from this review are presented using bibliometric and keyword network analyses. The results are used to propose a meta-framework that organizes the main research topics of PMSs in non-profit organizations and a framework to consolidate factors that influence the design-implementation aspects of PMSs in non-profit organizations.

## Performance Measurement Systems in Non-Profit Organizations

Moxham (2009, 2014) observes that there is no consensus or agreement about the definitional terminology for non-profit organizations. For example, the characteristics of a charity institution is usually related to a kind of non-profit oriented, but not all the NPO must be, of course, a charity organization. In this context, the sector is diversified, including cooperatives, voluntary agencies, religious institutions, hospitals, museums, trade unions, universities, civil rights groups, and third sector organizations. In this perspective of creating social value, it could be also added to the perspective of public administration within the NPO context (Karwan and Markland, 2006; Moxham, 2009, 2014; Sole and Schiuma, 2010; Valentinov, 2011).

Moura et al. (2019) explain how NPO and public administration pursue the social value creation for their audience instead of the financial profit. Both organizations have financial restrictions when compared to traditional enterprises, as well the involvement of stakeholders and management issues, such as short and long-term planning, fairness, accountability, and legitimacy. Although they have different legal characteristics (public administration works within a government plan context while NPO usually works using projects connected or not to public administration), aspects related to their performance measurement and management are similar and can be studied together (Conaty, 2012; Berman, 2014; Sinuany-Stern and Sherman, 2014; Moura et al., 2019).

For the purpose of this work, an NPO is defined as an organization with financial restrictions in that its surplus funds cannot be distributed or shared with those who control it, but which can be used for reinvesting in social targets (Moxham, 2009, 2014; Kong, 2010; Valentinov, 2011).

NPOs have characteristics that differentiate them from for-profit organizations, such as income sources that come from donations, private partnerships, or public investments; human resources as a working group of employees and volunteers; and accountability that requires transparency of financial accounts and resources to donors, investors, or regulatory agencies. According to Moxham (2009), trust and legitimacy are important features between NPOs and their stakeholders. For example, there has been increasing pressure by stakeholders for better accountability, especially when involving financial resources, such as donations. In this context, NPOs have gone through difficult and challenging times, and Kong (2010) points out that taxes, fees, decreasing tax incentives, governmental problems, and economic crises are examples of the challenges and barriers that an NPO must face.

For Moxham (2009) and Waal et al. (2011), there was no current answer for how to measure performance in NPO because the literature does not present a consensus about the criteria. First, because there is not enough research conducted on PMS design for NPO and second, it is difficult to measure performance results in NPO. Also, Arena et al. (2015) give other reasons that can be attributed to the lack of financial, human, and technological resources for PMS design-implementation aspects.

Pinheiro de Lima et al. (2008) and Waal (2007) describe a PMS as a set of processes that transforms mission, strategy, and organizational goals into key measurable performance indicators that govern organizational actions. Silvi et al. (2015) suggest PMS is considered strategic when it embeds characteristics for long and short term planning, financial and non-financial indicators, future perspectives, internal and external viewpoints, and includes causes and effects of relations between measures and system aspects.

Ospina et al. (2002) recognize that most of the tools and models for performance management have been developed considering for-profit companies. However, a PMS would be useful to non-profit organizations as well. For Austin (2000), the number of non-profit organizations is increasing, especially because of the growing number of complex social problems that need to be addressed. Also, political issues, legal obligations, and stakeholders' requirements have prompted some non-profit organizations to apply entrepreneurial strategies and business models to become more competitive and transparent.

According to Waal et al. (2011), implementing and using PMSs in the non-profit sector is more challenging as there is a relative lack of clarity in the purpose of the system in this kind of organization. Although there are many options of PMSs, few of them are designed for NPO. Usually, the available frameworks for NPO are adapted from the for-profit organizations, but they do not consider all their characteristics. There are public agencies working with some PMSs, but the adaptation for NPOs are usually flaw regarding, for instance, the use for decision making support.

Some examples of frameworks adapted for NPO can be mentioned. First, Lee and Moon (2008, p. 26) suggested “a BSC model of social enterprises in which social objectives are attained as a result of interrelationships between four perspectives: financial, customers (stakeholders), internal business process, and learning and growth.” The work of those authors focuses on how the BSC can be used in the context of an NPO. Also, Meadows and Pike (2009, p. 133) propose a Social Enterprise Scorecard based on adapting BSC “to make it more applicable to social enterprises.” They argue that “the scorecard needs to take a holistic view of organizational life, and the perspectives of a diverse group of stakeholders. Social return is the prime concern for social enterprises and must be emphasized.” Somers (2005, p. 48) proposes a Social Enterprise Balanced Scorecard (SEBC) and “to amend the original Kaplan and Norton Balanced Scorecard three changes were introduced: an additional layer was added in which social goals are articulated above the financial perspective; the financial perspective was broadened to focus on sustainability, and the customer perspective was widened to capture a larger number of stakeholder groups.” Arena et al. (2015, p. 668–669) propose a generic model for a Social Enterprise (SE) developing a PMS. The study “identifies what measurement dimensions are relevant for a SE (financial sustainability, efficiency, effectiveness, impact).” Sowa et al. (2004, p. 712) propose a model for NPO that considers the organizational effectiveness, named MIMNOE (Multidimensional and Integrated Model of Non-profit Organizational Effectiveness) that “captures two distinct levels or dimensions of effectiveness–management effectiveness and program effectiveness. Both management and program effectiveness are decomposed further into two subcomponents: capacity and outcomes.” Micheli and Kennerley (2005) investigated the adaptions of existing frameworks and case studies in NPO. Some examples of their findings are adaptations from the system theory, quality management, BSC, performance prism and the “Singapore quality award (SQA) model of business excellence with the BSC approach” (Micheli and Kennerley, 2005, p. 129). Furthermore, it is recognized that factors such as the lack of training, infrastructure, and flow of information hinder the effectiveness of a PMS in this type of organization (Micheli and Kennerley, 2005; Moxham, 2009, 2014; Strang, 2018). Cestari et al. (2018) propose a methodology that implements information extraction, organization, and analysis as a tool to document case studies of performance measurement system characteristics in NPOs. Moura et al. (2019) provide a conceptual framework that identifies and classifies the factors that influence the design of PMSs in NPOs and public administration. Moura et al. (2020) examine the design factors in PMSs in NPO and public administration exploring inter-relationship among them. Their results point to how these organizations have distinct differences compared to traditional businesses considering their organizational characteristics, complexity, and dynamics.

From the previous discussion, it is possible to notice that there is still some ground to be covered until a complete comprehension of PMSs for NPO is achieved. Specifically, guidelines for design, implementation, and use of PMSs for NPO must be identified and provided once their structure must be designed to be complex, in-depth, able to include all organizational characteristics, and for flexible interface considering the social goals and the management style (Peursem et al., 1995; Micheli and Kennerley, 2005).

## Research Design

Besides a meta-framework that organizes the main research topics related to PMSs in non-profit organizations, this paper also proposes a framework that groups factors that influence the design-implementation aspects of PMSs in non-profit organizations. At first, a literature review method is applied to map the body of knowledge of this field of study. Next, bibliometric, network and content analysis techniques are executed to describe current research themes and extract current information that could be used in the development of the mentioned meta-framework and the design-implementation framework.

To achieve this purpose, the research design of this work is organized in three main steps: (1) literature review; (2) the application of bibliometric, network, and content analysis techniques; and (3) the proposal of the (meta) framework.

In the first step, a literature review is applied to map the body of knowledge of this field and to generate significant information about PMSs in non-profit organizations. According to Tranfield et al. (2003), this method can provide a comprehensive map of the body of knowledge for a specific field, supports, and underpins the beginning of new academic research, since knowledge generated about this area could be mapped. Thus, it is particularly useful in exploratory research about incipient fields. Although this research did not follow specific frameworks for systematic literature review, some of the criteria included in PICO/PICOS, PEO, and SPIDER (Bowers et al., 2011) were adopted. Those techniques/frameworks help break down the research question into pertinent components and derive to search criteria (Methley et al., 2014), and their acronyms stand for the components they adopt to structure/develop the research questions. Table 1 exposes a brief comparison about those frameworks, based on Davies (2019).

**Table 1:** Frameworks that help the development of the research questions.

**Frameworks**	**Components (items)**	**Main use**
PICO/PICOS	**P**opulation/problem/ phenomenon **I**ntervention **C**omparison **O**utcome **S**tudy design	Evidence-based reviews comparing interventions on a population.
PEO	**P**opulation **E**xposure **O**utcome	Qualitative research questions.
SPIDER	**S**ample **P**henomenon of **I**nterest **D**esign **E**valuation **R**esearch type	Qualitative and mixed-methods research questions.

The process to identify a portfolio that addresses PM in NPOs was conducted by the literature review design described by Keathley (2016). The author describes the research about the factors that affect the successful implementation of PM systems, and her method carries to identify all relevant publications.

**Figure 1:**
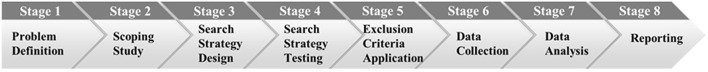
Literature review stages.

A set of procedures to guide the application of the literature review were chosen based on the characteristics and features exposed in Cochrane review handbook (Higgins and Green, 2011), and these procedures can be iterative and are organized in stages such as exposed in Figure 1.

The first stage is the problem definition, and it is described by the following research question: “What are the factors that influence the design and implementation processes of performance measurement systems in non-profit organizations?”

In the second stage, the scoping study, the researchers perform simple searches in databases and tests the search terms with simple Boolean phrases. In the current research, search terms in papers about PMSs in non-profit organizations were identified. The research question theme was used to determine the search terms of interest:

- Factors that influence the design and implementation.
- Performance Measurement Systems.
- Non-profit Organizations.

**Figure 2:**
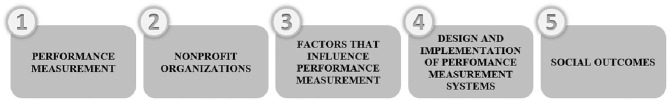
Groups of search terms.

A set of 20 papers is used as a thematic reference for the scoping study, that is, to refine the search string. As an iterative procedure, all papers from the control set are read in detail, and new search terms are identified. Eleven combinations of terms are tested resulting in five groups of search terms to compose the scoping study (presented in Figure 2).

The group of search terms supported the search strategy and then, they are applied to the Literature Review Protocol shown in Table 2, which contains search terms approved with the defined Boolean operators, chosen databases, language, and publication type. This way, many papers that are related to performance measurement, but did not cover the discussion of factors that influence design and implementation excluded from the search.

**Table 2:** Literature review protocol.

Search terms	Group 1: “performance management” OR “performance measurement” OR “performance indicators” OR “organizational performance” OR “performance metric” OR “non-financial performance measures” OR “performance measurement system” Group 2: “non profit organizations” OR “non-profit organizations” OR “not-for-profit organizations” OR “not for profit organizations” OR “not-for-profit organizations” OR “Non profit organizations” OR “Non-profit organizations” OR “not for profit organizations” OR “non profit service” OR “non-profit service” OR “not-for-profit service” OR “not for profit service” OR “voluntary organizations” OR “human service organizations” OR “non-governmental organizations” OR “voluntary organizations” OR “human service organizations” OR “non-governmental organizations” OR “social enterprises” OR “ngo” OR “npo” Group 3: barriers OR challenges OR competences OR characteristics OR components OR enablers OR motivations OR obstacles OR requirement Group 4: approach OR design OR framework OR method OR methodology OR process OR roles OR capabilities Group 5: “social care” OR “social goals” OR “social outcomes” OR “social activities” OR “social value” OR “social entrepreneurship” OR “social structure” OR “social work”
Boolean operator	AND among groups
Database	Science Direct, Emerald, Taylor and Francis, Scopus, Springer, Wiley, ISI Web of Science, Proquest
Language	English
Publication type	Academic journal papers

The next stages include data collection and exclusion criteria adoption. This search for papers resulted in a set of 4,606 papers in the data collection stage that results from the application of the “search expression.” After that, all abstracts of the retrieved papers were read, and only the papers that referred to non-profit organizations and performance measurement systems were kept in the paper set. A total of 310 papers were selected after the exclusion criteria adoption. Seventy of those papers were duplicates and, therefore, eliminated. This process resulted in a final paper set of 240 papers that covers journal papers from the past 30 years.

Following this, in the second step, techniques such as bibliometric analysis and keyword network analysis were applied to describe current research topics related to the theme. This combination of techniques contributed to consolidate the findings and highlight the significance of the results. The bibliometric analysis is based on the quantitative assessment of certain parameters for a defined set of papers, such as their authors, references, citations, and journals.

To execute the bibliometric analysis, the MC3R software (FLUXO Business Automation, 2015) were used to organize all dataset information in reports and matrices. The MC3R is a web-based platform that supports the development of literature review and its pertinent tasks. The 240 papers were uploaded into the software, including information such as paper title, publication year, authors and their countries, keywords, publication journal, cited references, and others.

After that, the whole dataset was double checked. Finally, the software provided reports which enable the characterization of the paper set, including the distribution of paper set and cited references per year, publication journals, journals from references, also, the most frequent authors and their countries, the keywords, and the cited references and their authors.

The data registered in MC3R software is also used to generate a keyword co-occurrence matrix. Then, UCINET software, as described by Borgatti et al. (2002), was used to construct a network of keywords and to obtain a network-related indexes' report. The frequency of keywords associations is calculated to construct maps (strategic diagrams) that represent the major themes of the field under study, and the relationships among them. Additionally, a k-core analysis was performed to represent a set of nodes that have connections to at least “K” other nodes in the set. The analysis is used to figure out the importance of a research topic in the network, and to delimit groups of similar research interests.

In the last step, all the findings related to bibliometric and network analyses are consolidated. Therefore, it is possible to propose a meta-framework that organizes the main research topics of PMS in non-profit organizations that can support future work and a framework to consolidate factors that influence the design-implementation stage of PMSs in non-profit organizations.

## Bibliometric and Network Analyses

The results of the bibliometric analysis are the paper set characterization, including distribution of papers and references, authors and their countries, cited authors, publications and journals, keywords analysis, and cited references.

The first set of analyses examined the distribution of the 240 papers from the portfolio distributed per year of publication. There is a generally increasing interest, since 2001, in the topic of non-profit organizations and PMSs. Afterward, a significant improvement was detected since 2007. This growth could justify the development of a local theory for performance measurement.

It is also perceived that as the knowledge of this research area is becoming specialized, the cited references tend to be more recent. Thus, the gap between the published articles and the cited references is reduced. Also, the area becomes more professional and begins generating specific knowledge in this field.

Another significant result of the bibliometric analysis was the keyword analysis. Papers in the paper set provided 615 keywords. The present analysis considers only terms that are separately identified in the papers under the label of “keywords.”

Of the 615 keywords, there are 501 that appear only once. That is, around 81% of the keywords proposed are cited only one time in the paper set. There is a meaningful evidence of terms usually related to PMSs, such as “performance measurement,” “performance management,” “balanced scorecard,” “performance,” “evaluation,” and “accountability.” This fact may suggest that PMSs are on the research agenda of non-profit organizations. Other keywords of this group, for instance, “social enterprise” and “social entrepreneurship” are used to define what type of non-profit organization is addressed in the paper.

The terms “balanced scorecard,” “evaluation,” and “accountability” are among the top 10 cited keywords indicating that they are closely related to research associated with performance measurement in non-profit organizations. Table 3 shows the most frequent keywords.

**Table 3:** Most frequent keywords.

**#**	**Keywords**	**Frequency**	**#**	**Keywords**	**Frequency**
1	Performance measurement	30	27	SROI	5
2	Performance management	22	28	Charity	4
3	Non-profit organization	21	29	Data envelopment analysis	4
4	Balanced scorecard	17	30	Efficiency	4
5	Social enterprise	15	31	Government	4
6	Non-profit	13	32	Health service	4
7	Performance	13	33	Local government	4
8	Evaluation	11	34	Public administration	4
9	Accountability	10	35	Public sector	4
10	Social entrepreneurship	10	36	Change management	3
11	Market orientation	9	37	Empowerment	3
12	United Kingdom	9	38	England	3
13	Third sector	8	39	Impact measurement	3
14	Non-governmental organization	7	40	Management	3
15	Performance measure	7	41	Measurement	3
16	Leadership	6	42	New public management	3
17	Organizational effectiveness	6	43	New Zealand	3
18	Organizational performance	6	44	Non-profit accountability	3
19	Outcome measurement	6	45	Policy	3
20	Public sector organizations	6	46	Public sector reform	3
21	Case study	5	47	Quality	3
22	Child welfare	5	48	Strategic management	3
23	Human service	5	49	The Netherlands	3
24	Outcomes	5	50	Transformational leadership	3
25	Social impact	5	51	Trust	3
26	Social value	5			

The term “accountability,” for example, shows the concern about stakeholders' requirements as legal obligations to provide financial and management reports. Accountability can contribute to reaching new investments and donors, in addition to providing information and legitimacy for funding and regulatory agencies. The term “SROI (Social Return on Investment)” appears as a new term and it indicates a performance measurement tool adapted for non-profit organizations to demonstrate the social and economic impact that they generate.

The results obtained as “accountability,” “leadership,” “social impact,” “efficiency,” and “quality” represent important findings and they indicate significant factors that influence performance measurement. Considering all the gathered information, a network of keywords was created using the UCINET® software (Borgatti et al., 2002).

**Figure 3:**
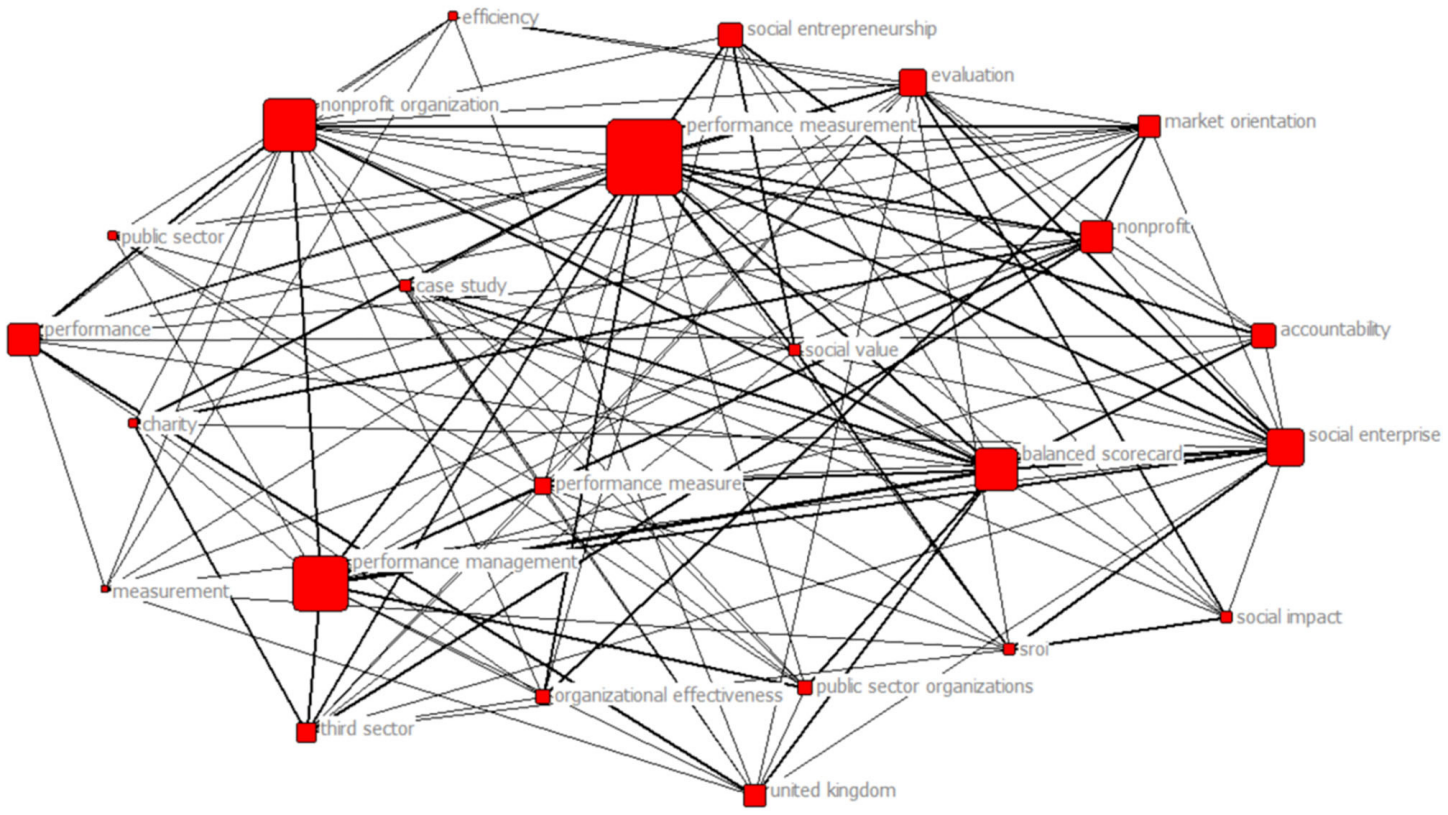
Seven-core keywords network.

Figure 3 shows the seven-core group network for the keywords from the documents in the paper set that appear at least three times. The size of each square indicates the frequency of each keyword. The thickness of the edges indicates the frequency with which two keywords were cited together.

The seven-core group is the most expressive of the network and includes the studies about performance measurement challenges in non-profit organizations. Also, studies show frameworks proposed for a balanced scorecard in non-profit organizations. Despite the increased adoption of the balanced scorecard methodology by numerous business organizations during the last decade, limited case studies are developed concerning non-profit organizations and their specificities (Grigoroudis et al., 2012).

An interesting finding is that the seven-core network also shows themes related to social aspects, such as “social impact,” “social value,” “social entrepreneurship,” and (social return on investment) “SROI” for example. In the literature, Wilson and Bull (2013) used SROI in a small social enterprise for measuring social impact. Moreover, SROI is a framework for understanding and measuring the social, economic, and environmental value of an organization's activities with a focus on outcomes, different from other tools in placing a monetary value on the outcomes and benefits.

The top three authors, R. Andrews (United Kingdom), R. M. Walker (China), and G. A. Boyne (United Kingdom) have jointly authored papers together. Of the four papers from R. M. Walker and G. A. Boyne, three of them are authored with R. Andrews.

Further analysis was conducted on cited authors. Papers in the paper set presented over 13,000 authors in the cited references. Eighty-five of them were referenced more than 10 times, and G. A. Boyne was the most cited author with 44 citations.

G. A. Boyne's papers have a focus on public administration and were published between 1996 and 2011. The next four authors deal with different contexts. L. Salamon's papers address non-profit organizations in general, public sector, third sector, and social welfare organizations. R. M. Walker performs research on social welfare organizations, the voluntary sector, and public organizations. R. S. Kaplan focuses on the Balanced Scorecard for any organization and the public sector. A. Neely's papers deal with performance measurement and management in general.

The next analysis considers journal publications. Firstly, it is important to notice that of the total of 136 publication journals of the papers set, “Voluntas: International Journal of Voluntary and Non-profit Organizations” and “Administration in Social Work” are the most frequent ones with 15 and 12 papers, respectively (10 journals in the paper set represent 32% of the total). Curiously, eight of them are journals with a public administration or non-profit subject as their focus of publication. In the paper set, only the “International Journal of Productivity and Performance Management” journal explicitly publishes papers related to performance management and measurement.

The most frequent journal appearing in the references of the paper set is “Non-profit and Voluntary Sector Quarterly” with 166 appearances, which was the fifth most frequent journal in the paper set. Of the 10 most frequent journals for the cited references, five of them have a focus on public administration or non-profit organizations, and eight of them have a high-level classification (Q1) by Scimago.

Finally, there are 10,540 cited references in the paper set and 9,136 of them (around 87%) are cited just once. Table 4 shows the 10 most cited references which the focus is “performance measurement” and, indeed, citations are mostly focused on two themes: “performance measurement systems” and “management of non-profit organization.”

**Table 4:** Most frequently cited references.

**#**	**References**	**Authors**	**Year**	**Citations**
1	The balanced scorecard - Measures that drive performance *Harvard Business Review, 70, 1, 71–79*	Kaplan, R. S.; Norton, D. P.	1992	28
2	The Balanced Scorecard – Translating Strategy into Action *Harvard Business School Press*	Kaplan, R. S.; Norton, D. P.	1996	25
3	Strategic Performance Measurement and Management in Non-profit Organizations *Non-profit Management and Leadership, 11(3):353–370*	Kaplan, R. S.	2001	24
4	Measuring the unmeasurable: Empirical studies of non-profit organization effectiveness *Non-profit and Voluntary Sector Quarterly, 27, 183–202*	Forbes, D. P.	1998	19
5	The iron cage revisited: Institutional isomorphism and collective rationality in organization fields *The University of Chicago Press, 63–82*	DiMaggio, P.; Powell, W.	1991	18
6	Managing and Measuring Social Enterprises *Sage Publications*	Paton, R.	2003	17
7	Multiple Constituencies and the Social Construction of Non-profit Organization Effectiveness *Non-profit and Voluntary Sector Quarterly, 26(2): 185–206*	Herman, R. D.; Renz, D. O.	1997	15
8	The Economics of Performance Management in Non-profit Organizations *Non-profit Management and Leadership, v. 13, n. 3, p. 267–281*	Speckbacher, G.	2003	15
9	Using the Balanced Scorecard as a Strategic Management System *Harvard Business Review, 74 (1), 75–85*	Kaplan, R. S.; Norton, D. P.	1996	15
10	Case Study Research: Design and Methods (2nd ed.) *Sage Publications*	Yin, R. K.	1994	15

Some classic references on performance measurement, such as those from Kaplan and Norton (1992) and Kaplan and Norton (1996), are the most cited in the paper set. These references are also some of the most popular when considering purely the field of performance measurement (Neely, 2005). It is noteworthy that, although the topics of performance measurement and non-profit organizations are addressed, this paper is not a result of the search, since it did not have keywords that addressed factors that influence the design-implementation of PMSs. Therefore, the knowledge of PMSs for for-profit organizations seems to be used as a foundation for research on PMSs for non-profit organizations. Indeed, as observed by Arena et al. (2015), this confirms what had already been pointed out: the simple adaptation of for-profit PMSs approaches to non-profit organizations appears not to be enough to address the characteristics of non-profit organizations.

Two of the references in Table 4, Forbes (1998) and Herman and Renz (1997) discuss the difficulty of measurement effectiveness in a non-profit organization. Forbes (1998) reviewed empirical studies of non-profit effectiveness from 1977 to 1997, while Herman and Renz (1997) investigated stakeholder judgments of non-profit charitable organization effectiveness. According to Forbes (1998), there are several concepts of effectiveness in non-profit organizations used by researchers.

Three of the references in Table 4 address performance measurement in a non-profit organization (Kaplan, 2001; Paton, 2003; Speckbacher, 2003). These works propose options for adapting the balanced scorecard to a non-profit organization and suggest that for-profit themes of performance management may apply to non-profit organizations.

Another key point concerning the references is the theoretical background that is employed. For this purpose, the 60 most cited references were analyzed and divided into three main groups: (i) references that present general themes; (ii) references that present specific themes that apply to non-profit organizations; and (iii) references that utilize both general and specific themes.

Ninety-two percent (92%) of the examined references mention general themes, around 68% highlight specific themes that apply to non-profit organizations, and 62% consider both. The most common background of general themes is “balanced scorecard,” “performance measurement,” and “accountability,” which are the same themes that emerged in previous analyses. Also, “institutional theory,” “theory of organization,” and “stakeholders” were cited in the building of the knowledge in this field.

Lynch-Cerullo and Cooney (2011) examined the field-level pressures facing humanitarian service organizations (HSO) and review the research on performance measurement among non-profit HSOs on responses to these pressures and proposed a conceptual framework combining institutional theory and resource dependency theory. Additionally, the factors that encouraged performance measurement in non-profit organizations are examined.

According to Herman and Renz (1999), many ideas first introduced and popularized in business are later adopted by NPO, such as strategic planning, total quality management, and others. The belief is that what works in business should also work in non-profit organizations or what is considered as best practices is a sign of effective management and could legitimize a non-profit organization from a stakeholder's perspective. Therefore, the study is based on general and specific literature on organizational effectiveness to present those aspects regarding the non-profit organization effectiveness. Table 5 exposes that the number of specific themes is significant.

**Table 5:** General and specific themes from most frequently cited references.

General themes	Accountability, Balanced Scorecard, Economic theory of the firm, Funding, Institutional theory, Legitimacy, Management control theory, Management Practices, Management system, Market orientation, Neo-institutional theory, Organization Effectiveness, Organization theories, Organizational change, Organizational Effectiveness, Organizational Learning, Organizational performance, Organizational strategy, Outcome Measurement, Performance, Performance management, Performance measurement, Performance measurement systems, Performance Measures, Reporting, Resources, Stakeholders, Strategy, Theory of organization
Specific themes	Categorization of non-profit organizations, Charitable organizations, Environmental and social impacts, Human service organizations, Government sector, Multidimensional and integrated model of non-profit organizational effectiveness (MIMNOE), Non-governmental organizations (NGOs), Non-profit organization (NPO) accountability, Non-profit organizational effectiveness, Non-profit organizations, Non-profit sector, Public sector, Social audit, Social change, Social constructionism, Social enterprise, Social entrepreneurship, Social mission, Social performance, Social value, Social return on investment (SROI), Social sector, Third sector, Voluntary sector

A good example regarding the specific literature is the Multidimensional and Integrated Model of Non-profit Organizational Effectiveness (MIMNOE) proposed by Sowa et al. (2004), which builds upon debates in organizational theory and non-profit management research and suggests a multidimensional model to capture non-profit organizational effectiveness.

## Discussion

The bibliometric and network analysis highlighted the main characteristics of performance measurement systems in non-profit organizations' research. In this section, findings from the works of the literature are discussed.

There are three focus areas to be highlighted. The first one is related to the diversity of non-profit organizations, of different types and with different concerns regarding performance.

The second one is the significant amount of works found in the systematic literature review that is related to performance measurement in non-profit organizations and that make use of the general body of knowledge in performance measurement.

Finally, such theories and models are the building blocks for the factors that influence different aspects of performance measurement systems.

### Types of Non-profit Organizations

In the literature, a significant variety of terms reflects the different typologies of non-profit organizations and appears as prevalent topics, like “third sector organization,” “non-governmental organization,” “civil society organization,” “public organization,” “social enterprise,” “social entrepreneurship,” “voluntary organization,” among others.

These organizations have the social aspect as a common goal, although they have specific aims and it reflects the difficulty to have measures that capture value across so many different organizations. Then, as mentioned by Moxham (2009), there is not an agreement about the terminology to “non-profit organizations,” which indicates that a charity institution is a kind of non-profit but not all organization have to be a charity organization. In this context, the sector is diversified and includes religious institutions, hospitals, museums, voluntary agencies, trade unions, universities, civil rights groups, cooperatives, and the third sector. Public administration appears in the literature review once, as already mentioned, it shares some characteristics with non-profit organizations as they play complementary and supplementary roles. There is not a consensus about the NPO terminology and which kind of organization can be included as one. Some works discuss NPO separated from the public sector or social enterprise (Karwan and Markland, 2006; Duque-Zuluaga and Schneider, 2008; Moxham, 2009).

For economic theories and models standpoint, Moxham (2009) and Valentinov (2011) take an NPO as having financial restriction about the profit sharing for investors or controllers. Also, this kind of organization depends on financing and donations. In this context, the requirements for these organizations may hinder organizational success.

### Models and Theories

Bibliometric, network, and content analysis revealed that several performance measurement theories and models are used to construct knowledge in this field. Theories such as “economic theory,” “institutional theory,” “organization theory,” “stakeholder theory,” “balanced scorecard,” amongst others, are frequently used and cited to support research in this area.

Steinberg (2003) evaluated economic theories of the non-profit sector to describe the sector, formulate governmental policy toward the sector and manage non-profit organizations. Then, the study presented theories' capacity to enlighten the understanding of inquiry, size, and scope of the sector, and the behavioral responses of donors, volunteers, paid staff, and non-profit organizations to changes in their external environment.

According to Brignall and Modell's (2000) studies in the public sector, the institutional theory has implications for the effective implementation of multidimensional performance measurement and management. Additionally, a proper definition suggested by institutional theory is that performance should be described as “institutionally” defined, that is performance-related factors determine the interests pursued by these organizations.

Herman and Renz (1999) studies draw from general and specific literature on organizational effectiveness to present propositions about non-profit organizations' effectiveness. They suggested that concerns about non-profit organization accountability, outcomes assessment, and performance evaluation confirm the relevance of the discussions about non-profit organizations' effectiveness. For Sowa et al. (2004), bearing in mind the organizational diversity, it is important that these differences must lead to the appropriate criteria for assessing effectiveness.

The identification of these theories in previous studies confirmed that research in this area builds upon general performance measurement research. Furthermore, as observed by Luke et al. (2013), it is essential to note that the “balanced scorecard” is the most cited model in the references and its importance is also concerned with the purpose of ensuring assessment of organizational performance outcomes and impact, besides legitimacy of the communication.

The balanced scorecard is a classic example of an adapted model from the general performance measurement field to non-profit organizations. Also, the performance prism model is another example of a performance measurement tool used in the for-profit sector that has been adapted to non-profit organizations (Lee and Moon, 2008; Meadows and Pike, 2009; Moxham, 2009; Mouchamps, 2014; Arena et al., 2015).

Somers (2005) suggests that the balanced scorecard needs to be adapted to the social enterprise by including social goals, expanding the financial perspective to emphasize sustainability and the customer perspective being widened to capture multiple stakeholders' perspectives. Her research presents that by using the Social Enterprise Balanced Scorecard (SEBS), organizations have positive outcomes and become a better business. Also, social enterprises that use this model can demonstrate social value added to stakeholders.

Moreover, there is an accounting terminology being disseminated to more efficiently evaluate and measure blended value creation in the third sector. Consequently, concepts such as SROI (Social Return on Investment), social accounting and audit, Social Return Ratio (SRR) were developed and reflect specific theories in this research area (Moxham, 2009; Luke et al., 2013).

Banke-Thomas et al. (2015) consider SROI as a model that can measure social and economic outcomes and analyzes views of different stakeholders in a monetary ratio through comparison between net benefits to the investment required. In other words, Wilson and Bull (2013) complement saying that SROI is a framework for understanding and measuring the social, economic, and environmental value of an organization's activities. Another example is the Social Accounting and Audit, as mentioned by Luke et al. (2013), which is an externally audited report of social value creation.

However, for many non-profit managers, performance management systems adapted from the private sector are seen with skepticism, as it is observed by Moxham (2009) and Straub et al. (2010). In this context, Moxham (2009) investigates the applicability of the existing body of knowledge about performance measurement in private and public sector non-profit organizations.

It is noteworthy that the research about performance measurement systems in non-profit organizations is gradually becoming specialized and has started to build upon prior research in the area. From this perspective, there are some examples of specific models and theories about performance measurement systems in non-profit organizations. An example of a specific model for a non-profit organization is the Multidimensional and Integrated Model of Non-profit Organizational Effectiveness (MIMNOE) proposed by Sowa et al. (2004) and previously presented. This framework builds upon discussions in organizational theory and non-profit management research and suggests a multidimensional model to capture non-profit organizational effectiveness.

### Factors That Influence the Design-Implementation of PMSs

The main factors that influence the design-implementation aspects of PMS for NPO need to be identified. For Micheli and Kennerley (2005) the number of frameworks is small yet so that investigations will be necessary for the research area. Some tools and methods have been developed, but as observed by Arena et al. (2015), the systematic analysis is not enough. The PMS evolution was not capable of knowing all various dimensions/factors about the performance in NPO. Understanding them will contribute to translating the social issues in measurable terms.

**Figure 4:**
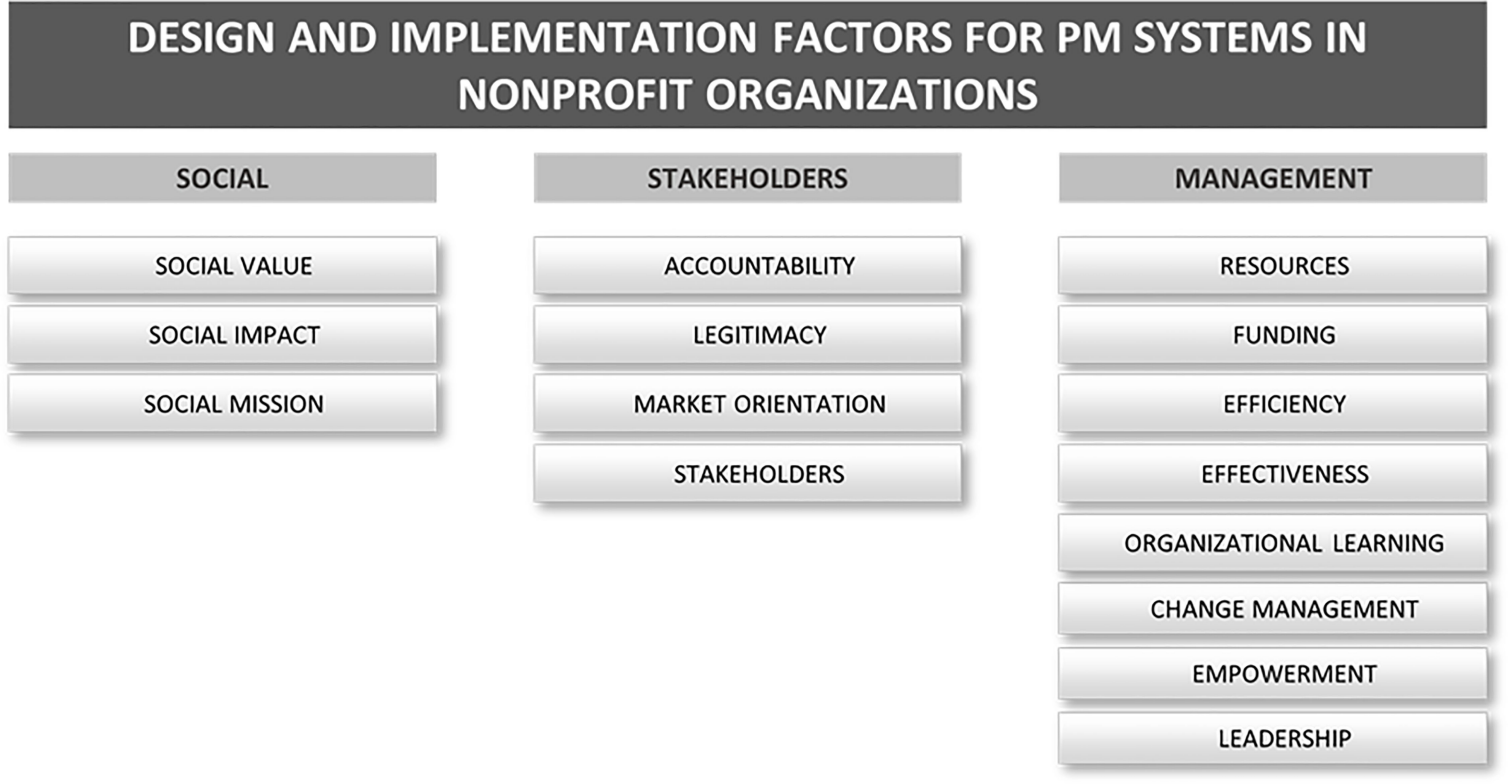
Framework for the factors that influence the design-implementation of performance measurement.

In this sense, Figure 4 depicts a framework that consolidates the main factors that influence the design-implementation aspects of performance measurement systems identified in the literature review performed in this work. Design-implementation factors are retrieved from a content analysis of the paper set, where the most frequent terms were grouped into three main categories: (i) social factors; (ii) stakeholder-related factors; and (iii) managerial factors.

Considering the social approach, it is important to mention that the desire for social results of these organizations must reflect the development of performance measures, the choice of performance measurement systems and must be in the organization's mission, vision and within the behavior and commitment of the people involved.

Therefore, in addition to the correct choice of measures and strategies, the PMS design-implementation process needs to consider the interaction of the employees and volunteers involved. In addition to these internal stakeholders, NPOs are highly involved with their external stakeholders, which leads to the relation of the second group of factors.

An NPO generally has legal or contractual financial restrictions. Accountability and the search for legitimacy is part of the routine and can not only be a legal obligation, but also as strategically interesting in attracting donors, financiers, and partnerships. In this sense, the choice of a PMS and its design-implementation process must consider ways to guarantee and to achieve factors related to accountability, legitimacy, market orientation, and stakeholders.

Regarding the managerial aspects, design-implementation factors of PMS present several attributes related to the management of the organization in terms of human resources, project, resources, and capability. Factors such as use and availability of financial resources and funding are conditions that affect investment, planning and management decisions in NPOs.

The stakeholder's demand for high levels of efficiency and effectiveness has pressured these organizations to seek measures and performance strategies to retain investors, seek new ones, transparency to the government and to their community. In addition, aspects related to organizational learning and empowerment are appearing more frequently as strategic points for managers who need to deal with paid employees, but also volunteers who are not acting for salary or professional growth, but are involved in these organizations by their beliefs, values and personal growth. How to motivate these people? How to control and measure the performance of these people within a context that considers social profit more relevant than the professional?

Factors in the social category represent the concern of non-profit organizations in achieving their social objectives and purposes. In this context, the measurement of performance in NPO is dependent on their aims, mission, and goals (Clarkson, 1995; Luke et al., 2013). For this reason, the social category, which includes “social value,” “social impact,” and “social mission” is a predominant topic in performance measurement for NPO. Also, Luke et al. (2013) suggested that differently from for-profit organizations that have profitability as a primary purpose, the underpinning objective of this kind of organization is to be financially viable such that they can continue to pursue their social mission. Furthermore, Costa et al. (2011) reported that the long-term performance of non-profit organizations concerns their capacity to expand social value as defined in their mission.

Complementary, stakeholder-related factors reflect the importance of different groups of stakeholders to non-profit organizations, particularly the necessity to fulfill their requirements. Mano (2013) indicates that NPO must present regular and reliable reports to stakeholders mainly on the reach of social goals within the restrictions of the funding and resources provided. In this regard, transparency to stakeholders, including measures of performance is also expected. According to Costa et al. (2011), non-profit organizations have emerged as significant actors for promoting social values. This increasing importance and influence have heightened requirements for more legitimacy and accountability, both internally and externally. In doing so, stakeholders can assess the impact of the activities developed by non-profit organizations. Nevertheless, non-profit “accountability” and performance measurement systems are usually more complex than those in for-profit companies, which focus on profit maximization and stockholders/shareholders as primary stakeholders. On the other hand, non-profit organizations have a socially oriented and ethically based mission and deal with multiple and competing stakeholder demands. Non-profits' financial sustainability does not guarantee the achievement of the organizational mission and several studies suggest that there is a strong relationship between “market orientation” and organizational performance for non-profit organizations (Duque-Zuluaga and Schneider, 2008; Walker et al., 2011).

Factors in the managerial category reflect the concerns of non-profit organizations to operationalize their activities so that their social objectives are fulfilled, as well as the requirements of their stakeholders. In this context, an important issue and prevalent topic is the dependence of non-profit organizations on “resources” and “funding.” Moreover, the competition for financial resources to fund non-profit services is intense. As observed by Moxham (2010), the provision of funding is dramatically decreasing. Similarly, Kaplan (2001) emphasizes the theme of accountability and performance measurement as urgent for non-profit organizations due to the increasing competition for “funding.” Consequently, as clearly stated by Hodge and Piccolo (2005), to secure “funding,” non-profits are under pressure to demonstrate “value for money.” In this context, non-profit organizations have a constant concern to measure their performance to satisfy stakeholders' expectations and consequently, to ensure their strategy in approaching “funding” and “resources” allocation and utilization. Similarly, “evaluation” is also a relevant topic and is directly related to “efficiency” and “effectiveness.” NPO should have approaches to performance evaluation that effectively capture both financial and social dimensions, which is crucial to demonstrate organizational legitimacy, transparency, credibility and to acknowledge the extent of their impact. According to Costa et al. (2011), because it is difficult to define clear key success performance indicators in NPO, it is also challenging to identify systems that can report to internal and external stakeholders on organizational “efficiency” and “effectiveness”–in other words, the extent to which organizations achieve their goals.

According to Bradshaw (2009), NPOs' council and boards must implement change management processes that can be used to orient them in reflecting on their choices related to governance frameworks, indicating what contingency factors should be considered. Change management strategies, as compiled by Herman and Renz (1998), could cover aspects such as legitimation, retrenchment, and new revenue strategies.

Leadership could be approached in the support provided by the board of directors to both initiatives related to change, and the implementation of performance measurement systems. Harrison and Murray (2012) recognized that boards of directors have a considerable impact on the performance of non-profit organizations, their CEOs, and on the support of key stakeholders. Their leadership position could be used to build high-quality relationships. Becker et al. (2011), shows that implementation of performance measurement systems required not only the technical system to be successful but also the support of senior management, with a strong commitment to development and implementation that facilitates a higher level of ownership and accountability for all involved actors.

Wellens and Jegers (2014) show that there is a consensus on the importance of an employee-organization fit. Particularly to volunteers, empowerment, quality of intra-organizational relationships and training, and support seem to be important. Employees' empowerment can be achieved through formal and informal mechanisms at different levels, such as personal job involvement and participation in overall organizational policymaking.

In summary, change management provided the meta-framework for discussing performance measurement system implementation in a non-profit organization, that requires leadership from the top level as well as from the team that is in charge of the implementation process. Empowerment will give the involved actors autonomy for experimenting and customizing models according to contingencies.

## Conclusion

This research provided a comprehensive synthesis of the study of performance measurement systems in non-profit organizations based on a literature review, bibliometric, and network analyses. A paper set with 240 articles related to the research field was examined. A large set of techniques was adopted to consolidate knowledge about this area of research. The present study makes several contributions to identifying the topics that are the most prevalent in this research area and their interrelationships. Furthermore, the findings enhance understanding of the extent that this area builds upon prior research. It is important to observe that public administration is identified in the review, as a complementary role in providing social value to society.

According to the results, it is possible to conclude that the investigation on performance measurement in non-profit organizations is still in its early stages of development with many opportunities to further develop the field. Although PMSs are a consolidated topic, the design-implementation of PMSs in non-profit organizations is a recent issue, and public administration studies reveal more maturity in managing through measures. Moreover, the results of this study suggest that, while there is significant interest in this research area, there are still many inconsistencies in the literature such as the terminology and the typologies used to refer to non-profit organizations.

In some cases, it was detected that a public administration perspective is strongly related to the studies of performance on non-profit organizations. In this sense, the research theme encompasses some works on public administration as they share some common characteristics with non-profit organizations.

Additionally, PMSs for non-profit organizations seem to be more complex than for for-profit companies, mainly because while the mission of for-profit companies is primarily to focus on profit maximization, non-profit organizations have an orientation for social mission and values. Also, NPO must deal with multiple stakeholders' demands and its financial sustainability does not guarantee the achievement of the organizational mission.

Thus, PMSs for non-profit organizations should include not only organizational viability but also the social impact of the organization. So, it is necessary that the development of PMSs frameworks, tools, processes, requirements, and indicators that address these specific features of non-profit organizations and consider multiple stakeholder perspectives.

Conceptual frameworks and models, as well as specific theories, are being generated for this field of research, and the process of adapting models from the general field of performance measurement is taking place. The meta-framework that organizes the main research topics of PMS in non-profit organizations and the framework that consolidates factors that influence the design-implementation of PMSs in non-profit organizations developed from the literature review represents a fundamental contribution to this field of study.

While this review is designed to be as comprehensive as possible, the main limitation of this approach is that the results are limited to the publications available on the searched platforms. As future work, it is recommended that a research agenda is structured for PMSs in non-profit organizations, identifying the main research groups and the main questions to be studied to contribute to the consolidation of research in this area of study. It is also suggested to include some sort of geographical analysis to understand and to identify possible patterns for developing the management systems. Besides that, the design-implementation factors identified in the literature review and part of the framework presented in this paper need further detailing using a more specific content analysis of the papers, as well as the development and analysis of case studies that can consolidate the application of these factors in non-profit organizations.

## Data Availability Statement

The original contributions presented in the study are included in the article, further inquiries can be directed to the corresponding author.

## Author Contributions

FT: overall methodology design for integrating portfolio formation and content analysis. LM: portfolio formation for design factors, content analysis, and models. JA: content analysis techniques selection, content analysis, overall review, and edition. EP: theoretical background for performance measurement in non-profit organizations and public administrations. FD: systematic literature review process design. SG: contributions assessment (theory, literature review method, and NPO/public administration practice). EV: paper review and performance measurement expert. JM: portfolio formation for implementation factors. LL: paper review and SLR expert. All authors contributed to the article and approved the submitted version.

## Conflict of Interest

The authors declare that the research was conducted in the absence of any commercial or financial relationships that could be construed as a potential conflict of interest.

## References

[B1] Arena M. Azzone G. Bengo I. (2015). Performance measurement for social enterprises. Int. J. Volunt. Nonprofit Organ. 26, 649–672. 10.1007/s11266-013-9436-8

[B2] Austin J. E. (2000). Strategic collaboration between nonprofits and businesses. Nonprofit Volunt. Sect. Q. 29, 69–97. 10.1177/0899764000291S004

[B3] Banke-Thomas A. O. Madaj B. Charles A. Van den Broek N. (2015). Social Return on Investment (SROI) methodology to account for value for money of public health interventions: a systematic review. BMC Public Health 15:582. 10.1186/s12889-015-1935-7 26099274 PMC4477315

[B4] Becker K. Antuar N. Everett C. (2011). Implementing an employee performance management system in a nonprofit organization. Nonprofit Manage. Leadersh. 21, 255–271. 10.1002/nml.20024

[B5] Berman M. (2014). Productivity in Public and NonProfit Organizations, 2nd Edn. New York, NY: Routledge 10.4324/9781315701509

[B6] Borgatti S. P. Everett M. G. Freeman L. C. (2002). Ucinet for Windows: Software for Social Network Analysis. Boston, MA: Harvard Analytic Technologies.

[B7] Bowers D. House A. Owens D. (2011). Getting Started in Health Research. Oxford: Wiley-Blackwell 10.1002/9781444341300

[B8] Bradshaw P. (2009). A contingency approach to nonprofit governance. Nonprofit Manage. Leadersh. 20, 61–81. 10.1002/nml.241

[B9] Brignall S. Modell S. (2000). An institutional perspective on performance measurement and management in the new public sector. Manage. Account. Res. 11, 281–306. 10.1006/mare.2000.0136

[B10] Cestari J. M. A. P. de Lima E. P. Deschamps F. Aken E. M. V. Treinta F. T. Moura L. F. (2018). A case study extension methodology for performance measurement diagnosis in nonprofit organizations. Int. J. Prod. Econ. 203, 225–238. 10.1016/j.ijpe.2018.06.018

[B11] Clarkson M. B. E. (1995). A stakeholder framework for analyzing and evaluating corporate social performance. Acad. Manage. Rev. 20, 92–117. 10.5465/amr.1995.9503271994

[B12] Conaty F. J. (2012). Performance management challenges in hybrid NPO/public sector settings: an Irish case, edited by Cláudia S. Sarrico. Int. J. Prod. Perform. Manage. 61, 290–309. 10.1108/17410401211205650

[B13] Costa E. Ramus T. Andreaus M. (2011). Accountability as a managerial tool in non-profit organizations: evidence from Italian CSVs. Int. J. Volunt. Nonprofit Organ. 22, 470–493. 10.1007/s11266-011-9183-7

[B14] Davies A. (2019). Carrying out systematic literature reviews: an introduction. Br. J. Nurs. 28, 1008–1014. 10.12968/bjon.2019.28.15.1008 31393770

[B15] Duque-Zuluaga L. C. Schneider U. (2008). Market orientation and organizational performance in the nonprofit context: exploring both concepts and the relationship between them. J. Nonprofit Public Sect. Mark. 19, 25–47. 10.1300/J054v19n02_02

[B16] Forbes D. P. (1998). Measuring the unmeasurable: empirical studies of nonprofit organization effectiveness from 1977 to 1997. Nonprofit Volunt. Sect. Q. 27, 183–202. 10.1177/0899764098272005

[B17] Frumkin P. (2005). On being Nonprofit: A Conceptual and Policy Primer. Cambridge, MA: Harvard University Press.

[B18] Grigoroudis E. Orfanoudaki E. Zopounidis C. (2012). Strategic performance measurement in a healthcare organisation: a multiple criteria approach based on balanced scorecard. Omega 40, 104–119. 10.1016/j.omega.2011.04.001

[B19] Harrison Y. D. Murray V. (2012). Perspectives on the leadership of chairs of nonprofit organization boards of directors: a grounded theory mixed-method study. Nonprofit Manage. Leadersh. 22, 411–437. 10.1002/nml.21038

[B20] Herman R. D. Renz D. O. (1997). Multiple constituencies and the social construction of nonprofit organization effectiveness. Nonprofit Volunt. Sect. Q. 26, 185–206. 10.1177/0899764097262006

[B21] Herman R. D. Renz D. O. (1998). Nonprofit organizational effectiveness: contrasts between especially effective and less effective organizations. Nonprofit Manage. Leadersh. 9, 23–38. 10.1002/nml.9102

[B22] Herman R. D. Renz D. O. (1999). Theses on nonprofit organizational effectiveness. Nonprofit Volunt. Sect. Q. 28, 107–126. 10.1177/0899764099282001

[B23] Higgins J. Green S. (2011). Cochrane Handbook for Systematic Reviews of Interventions, Version 5.1.0. Available online at: https://handbook-5-1.cochrane.org (accessed June 20, 2020).

[B24] Hodge M. M. Piccolo R. F. (2005). Funding source, board involvement techniques, and financial vulnerability in nonprofit organizations dependence. Nonprofit Manage. Leadersh. 16, 171–191. 10.1002/nml.99

[B25] Hoque Z. (2014). 20 years of studies on the balanced scorecard: trends, accomplishments, gaps and opportunities for future research. Br. Account. Rev. 46, 33–59. 10.1016/j.bar.2013.10.003

[B26] Kaplan R. S. (2001). Strategic performance measurement and management in nonprofit organizations. Nonprofit Manage. Leadersh. 11, 353–370. 10.1002/nml.11308

[B27] Kaplan R. S. Norton D. P. (1992). The balanced scorecard - measures that drive performance. Harvard Bus. Rev. 70, 71–79. 10119714

[B28] Kaplan R. S. Norton D. P. (1996). Using the balanced scorecard as a strategic management system. Harv. Bus. Rev. 74, 75–86.

[B29] Karwan K. R. Markland R. E. (2006). Integrating service design principles and information technology to improve delivery and productivity in public sector operations: the case of the South Carolina DMV. J. Oper. Manage. 24, 347–362. 10.1016/j.jom.2005.06.003

[B30] Keathley H. R. (2016). Empirical Investigation of Factors that Affect the Successful Implementation of Performance Measurement Systems. Montgomery, VA: Virginia Polytechnic Institute and State University.

[B31] Kong E. (2010). Analyzing BSC and IC's usefulness in nonprofit organizations. J. Intellect. Cap. 11, 284–304. 10.1108/14691931011064554

[B32] Lee Y. T. Moon J.-Y. (2008). An exploratory study on the balanced scorecard model of social enterprise. Asian J. Qual. 9, 11–30. 10.1108/15982688200800014

[B33] Luke B. Barraket J. Eversole R. (2013). Measurement as legitimacy versus legitimacy of measures: performance evaluation of social enterprise. Qual. Res. Account. Manage. 10, 234–258. 10.1108/QRAM-08-2012-0034

[B34] Lynch-Cerullo K. Cooney K. (2011). Moving from outputs to outcomes: a review of the evolution of performance measurement in the human service nonprofit sector. Adm. Soc. Work 35, 364–388. 10.1080/03643107.2011.599305

[B35] Mano R. (2013). Performance gaps and change in Israeli nonprofit services: a stakeholder approach. Adm. Soc. Work 37, 14–24. 10.1080/03643107.2011.637664

[B36] Meadows M. Pike M. (2009). Performance management for social enterprises. Syst. Pract. Action Res. 23, 127–141. 10.1007/s11213-009-9149-5

[B37] Methley A. M. Campbell S. Chew-Graham C. Mc Nally R. Cheraghi-Sohi S. (2014). PICO, PICOS and SPIDER: a comparison study of specificity and sensitivity in three search tools for qualitative systematic reviews. BMC Health Serv. Res. 14:579. 10.1186/s12913-014-0579-0 25413154 PMC4310146

[B38] Micheli P. Kennerley M. (2005). Performance measurement frameworks in public and non-profit sectors. Prod. Plann. Control 16, 125–134. 10.1080/09537280512331333039

[B39] Mouchamps H. (2014). Weighing elephants with kitchen scales: the relevance of traditional performance measurement tools for social enterprises. Int. J. Prod. Perform. Manage. 63, 727–745. 10.1108/IJPPM-09-2013-0158

[B40] Moura L. F. de Lima E. P. Deschamps F. Aken E. M. V. da Costa S. E. G. Treinta F. T. (2019). Designing performance measurement systems in nonprofit and public administration organizations. Int. J. Prod. Perform. Manage. 68, 1373–1410. 10.1108/IJPPM-06-2018-0236

[B41] Moura L. F. Pinheiro de Lima E. Deschamps F. M. Van Aken E. Gouvea Da Costa S. E. Tavares Treinta F. (2020). Factors for performance measurement systems design in nonprofit organizations and public administration. Meas. Bus. Excellence 24, 377–399. 10.1108/MBE-10-2019-0102

[B42] Moxham C. (2009). Performance measurement - examining the applicability of the existing body of knowledge to nonprofit organisations. Int. J. Oper. Prod. Manage. 29, 740–763. 10.1108/01443570910971405

[B43] Moxham C. (2010). Help or hindrance? Public Perform. Manage. Rev. 33, 342–354. 10.2753/PMR1530-9576330302

[B44] Moxham C. (2014). Understanding third sector performance measurement system design: a literature review. Int. J. Prod. Perform. Manage. 63, 704–726. 10.1108/IJPPM-08-2013-0143

[B45] Neely A. (2005). The evolution of performance measurement research: developments in the last decade and a research agenda for the next. Int. J. Oper. Prod. Manage. 25, 1264–1277. 10.1108/01443570510633648

[B46] Ospina S. Diaz W. O'Sullivan J. F. (2002). Negotiating accountability: managerial lessons from identity-based nonprofit organizations. Nonprofit Volunt. Sect. Q. 31, 5–31. 10.1177/0899764002311001

[B47] Paton R. (2003). Managing and Measuring Social Enterprises. London: Sage.

[B48] Peursem K. A. Van Lawrence S. R. Pratt M. (1995). Health management performance: a review of measures and indicators. Account. Aud. Account. J. 8, 34–70. 10.1108/09513579510103254

[B49] Pinheiro de Lima E. Gouvea da Costa S. E. Angelis J. J. (2008). The strategic management of operations system performance. Int. J. Bus. Perform. Manage. 10, 108–132. 10.1504/IJBPM.2008.015924

[B50] Silvi R. Bartolini M. Raffoni A. Visani F. (2015). The practice of strategic performance measurement systems: models, drivers and information effectiveness. Int. J. Prod. Perform. Manage. 64, 194–227. 10.1108/IJPPM-01-2014-0010

[B51] Sinuany-Stern Z. Sherman H. D. (2014). Operations research in the public sector and nonprofit organizations. Ann. Oper. Res. 221, 1–8. 10.1007/s10479-014-1695-2

[B52] Sole F. Schiuma G. (2010). Using performance measures in public organisations: challenges of Italian public administrations. Meas. Bus. Excell. 14, 70–84. 10.1108/13683041011074227

[B53] Somers A. B. (2005). Shaping the balanced scorecard for use in UK social enterprises. Soc. Enterp. J. 1, 43–56. 10.1108/17508610580000706

[B54] Sowa J. E. Selden S. C. Sandfort J. (2004). No longer unmeasurable? A multidimensional integrated model of nonprofit organizational effectiveness. Nonprofit Volunt. Sect. Q. 33, 711–728. 10.1177/0899764004269146

[B55] Speckbacher G. (2003). The economics of performance management in nonprofit organizations. Nonprofit Manage. Leadersh. 13, 267–281. 10.1002/nml.15

[B56] Steinberg R. (2003). Economic theories of nonprofit organizations, in The Study of the Non-profit Enterprise, eds Anheier H. K. Ben-Ner A. (Boston, MA: Springer), 53–65. 10.1007/978-1-4615-0131-2_16

[B57] Strang K. D. (2018). Strategic analysis of CSF's for not-for-profit organizations. Meas. Bus. Excell. 22, 42–63. 10.1108/MBE-07-2016-0035

[B58] Straub A. Koopman M. Van Mossel H.-J. (2010). Systems approach and performance measurement by social enterprises. Facilities 28, 321–331. 10.1108/02632771011031547

[B59] Tranfield D. Denyer D. Smart P. (2003). Towards a methodology for developing evidence-informed management knowledge by means of systematic review. Br. J. Manage. 14, 207–222. 10.1111/1467-8551.00375

[B60] Valentinov V. (2011). The meaning of nonprofit organization: insights from classical institutionalism. J. Econ. Issues 45, 901–916. 10.2753/JEI0021-3624450408

[B61] Waal A. (2007). Strategic Performance Management: A Managerial and Behavioural Approach. Basingstoke: Palgrave Macmillan.

[B62] Waal A. De Goedegebuure R. Geradts P. (2011). The impact of performance management on the results of a non-profit organization. Int. J. Prod. Perform. Manage. 60, 778–796. 10.1108/17410401111182189

[B63] Walker R. M. Brewer G. A. Boyne G. A. Avellaneda C. N. (2011). Market orientation and public service performance: new public management gone mad? Public Adm. Rev. 71, 707–717. 10.1111/j.1540-6210.2011.02410.x

[B64] Wellens L. Jegers M. (2014). Effective governance in nonprofit organizations: a literature based multiple stakeholder approach. Eur. Manage. J. 32, 223–243. 10.1016/j.emj.2013.01.007

[B65] Wilson D. Bull M. F. (2013). SROI in practice: the Wooden Canal Boat Society. Soc. Enterp. J. 9, 315–325. 10.1108/SEJ-03-2013-0013

